# Finerenone for diabetic hearts: a critical appraisal of MACE benefits and the research road ahead

**DOI:** 10.1093/ehjcvp/pvaf086

**Published:** 2025-12-16

**Authors:** Weikai Dong, Depin Li, Jie Zhao

**Affiliations:** Cardiovascular Medical Center, Nanjing Drum Tower Hospital, the Affiliated Hospital of Nanjing University Medical School, Nanjing 210008, Jiangsu Province, China; Department of Pharmacy, Nanjing Drum Tower Hospital, Affiliated Hospital of Medical School, Nanjing University, Nanjing 210008, Jiangsu Province, China; Cardiovascular Medical Center, Nanjing Drum Tower Hospital, the Affiliated Hospital of Nanjing University Medical School, Nanjing 210008, Jiangsu Province, China

We have read with great interest the recent publication by Chen *et al*.^1^ entitled ‘Nonsteroidal mineralocorticoid receptor antagonists and cardiovascular events in Type 2 diabetes: a retrospective study’. In this study, cardiovascular outcomes in patients with Type 2 diabetes mellitus (T2DM) treated with nonsteroidal mineralocorticoid receptor antagonists (MRAs), finerenone, as compared with spironolactone and eplerenone were explored. The authors should be congratulated for their use of a large real-world dataset, propensity score matching (PSM), and clinically relevant endpoints—major adverse cardiovascular events (MACE), mortality, and heart failure. However, we believe several aspects of the study merit further scrutiny and suggest directions for future improvement.

Firstly, the authors have performed PSM over clinical variables, but neither medication adherence nor baseline ejection fraction are included. As previously published registry-based data strongly indicate that these can significantly influence observed outcomes, particularly in medicated patients with heart failure,^2^ the balance of finerenone superiority may be confounded by unmeasured differences in baseline clinical status when not adjusted for treatment persistence or heart failure phenotype (i.e. HFrEF vs. HFpEF).

Secondly, the follow-up of this study (mean up to 24 months) is quite limited in comparison with the chronic nature of cardiovascular and renal disease in T2DM. Most of the adverse outcomes are late events which are usually beyond the follow-up. In this regard, results from FIGARO-DKD and FIDELIO-DKD (median follow-up 3.4 and 2.6 years, respectively) studies have shown that finerenone reduces the cardiovascular risk in patients with Type 2 diabetes with atherosclerotic cardiovascular disease over different time frames.^3^

Thirdly, although finerenone showed significantly greater reductions in MACE, mortality, and heart failure vs. spironolactone and eplerenone, the size of the effect was markedly reduced in subgroup analyses among women and patients with preserved renal function. This may represent a ceiling effect of finerenone, or it may indicate that the relative benefit of finerenone may be greatest in higher-risk phenotypes. Interestingly, a FIDELITY pooled analysis suggested that the mortality benefit of finerenone was predominantly seen among patients with advanced kidney disease and high albuminuria, a finding that underscores the importance of fine stratification by renal status and proteinuria.^4^

Furthermore, although the authors analysed laboratory covariates such as serum potassium and estimated glomerular filtration rate (eGFR), they did not analyse safety outcomes such as hyperkalaemia discontinuation or renal function decline, very closely. Comparative analyses have shown that finerenone would reduce the incidence of hypokalaemia as compared with steroidal MRAs, a beneficial quality for any long-acting therapy, particularly diabetics with renal disease.^5^

In conclusion, this study provides valuable real-world evidence for cardiovascular benefits of finerenone in T2DM. To increase clinical relevance of our results in future, studies should aim at including metrics of adherence, longer follow-up and more comprehensive analyses of safety. Prospective studies with neurohormonal biomarker data and echocardiographic parameters should explore whether it is mainly specific patient subgroups who obtain a net benefit. We offer suggestions aimed at refining an already exceptional article and eagerly anticipate future insightful contributions from the authors.

## Data Availability

No new data were generated or analysed in support of this research.
